# Intermittent Infusion Hemodiafiltration: A Narrative Review of an Emerging Dialysis Modality

**DOI:** 10.3390/toxins17090442

**Published:** 2025-09-03

**Authors:** Xiaoxi Zhou, Jing Sun, Lining Miao

**Affiliations:** 1 The Second Clinical Medical College, Jilin University, Changchun 130000, China; xxzhou18@163.com (X.Z.); s_jing@jlu.edu.cn (J.S.); 2 Department of Nephropathy, The Second Hospital of Jilin University, Changchun 130000, China

**Keywords:** intermittent infusion hemodiafiltration, end-stage renal disease, dialysis, uremic toxins, hemodynamic stability, individualized dialysis therapy

## Abstract

The number of patients with end-stage renal disease continues to grow worldwide, placing increasing demands on dialysis technologies. Conventional hemodialysis remains the dominant modality but is often limited by frequent intradialytic hypotension and the insufficient removal of medium-sized toxins. Intermittent infusion hemodiafiltration (I-HDF) is an emerging, hybrid dialysis technique that combines standard hemodialysis with the cyclic backfiltration of ultrapure dialysate. This approach enables dynamic blood volume control and periodic backflushing of the dialyzer membrane. Recent clinical studies demonstrate that I-HDF can reduce intradialytic hypotension incidence, improve systemic and microcirculatory perfusion, and enhance the clearance of middle molecules such as β_2_-microglobulin, while minimizing albumin loss. These benefits are particularly relevant to toxin clearance and hemodynamic stabilization, key priorities in optimizing dialysis outcomes. Large-scale cohort data suggest that I-HDF may be linked to improved long-term survival in dialysis patients. Given its physiological advantages and operational flexibility, I-HDF may also offer a practical solution in healthcare systems with limited access to high-volume online hemodiafiltration or kidney transplantation. Further research is warranted to develop individualized infusion protocols and validate its broader applicability.

## 1. Introduction

Kidney failure has become a growing public health concern worldwide. It significantly increases the incidence and mortality of related diseases and leads to rising medical costs [1]. The number of patients who rely on dialysis for survival continues to increase, with a global dialysis prevalence of 823 cases per million population [2]. Among these, hemodialysis (HD) accounts for approximately 89% of all dialysis treatments [3]. Under these circumstances, improving and developing dialysis technology is essential for enhancing patients’ quality of life and long-term outcomes.

However, conventional HD faces two significant clinical challenges. First, the high incidence of intradialytic hypotension (IDH) [4] disrupts dialysis sessions and increases the risk of organ hypoperfusion [5]. Second, it has a limited capacity to effectively remove middle-molecular-weight uremic toxins, which are associated with chronic complications and poor prognosis [6,7,8,9].

To overcome these limitations, intermittent infusion hemodiafiltration (I-HDF) has been introduced as a novel dialysis modality [10]. This technique combines standard HD with cyclic dialysate backfiltration, forming a convective approach that helps restore intravascular volume and clear middle-molecular-weight toxins through dialyzer membrane backflushing. Clinical use in countries like Japan shows practical value: by 2022, over 55,000 patients—more than 17% of the dialysis population—were receiving I-HDF [11].

This narrative review integrates clinical trials, cohort studies, and mechanistic analyses published between 2013 and 2025. It aims to provide a comprehensive summary of I-HDF’s technical implementation, physiological mechanisms, clinical benefits, and current limitations. In addition, it outlines potential future directions, such as the development of individualized infusion strategies to advance precision dialysis.

## 2. Mechanistic Basis of I-HDF

In I-HDF, a modest amount of fluid is intermittently infused into the bloodstream throughout the dialysis session [12]. A classical protocol administers approximately 200 mL of fluid every 30 min at a rate of 150 mL/min over about 80 s. Over a standard 4 h treatment, this results in seven infusions and a total substitution volume of approximately 1.4 L [13]. To maintain fluid balance, each infusion is followed by an equivalent amount of ultrafiltration during the next interval.

There are several ways to achieve fluid reinfusion in I-HDF [14]. These include using saline solution, sterile substitution fluid, or ultrapure dialysate delivered rapidly through machine settings. However, the most widely adopted method in current clinical practice is the backfiltration of ultrapure dialysate directly across the dialyzer membrane, which does not require additional infusate bags and integrates seamlessly into routine dialysis workflows. In published studies, a saline solution is typically used in protocols involving manual infusion, while studies utilizing automated or programmed infusion predominantly rely on the ultrapure dialysate.

**Figure 1:**
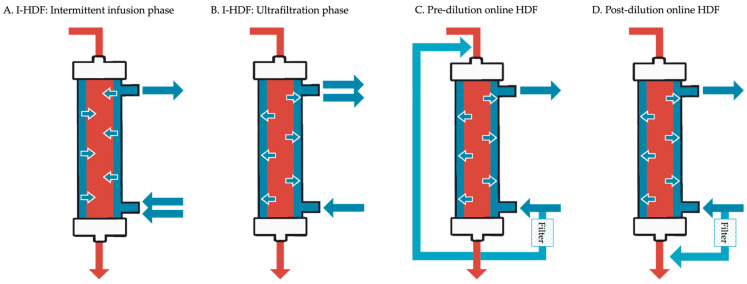
Schematic comparison of intermittent infusion hemodiafiltration (I-HDF) and online hemodiafiltration (HDF).

Unlike conventional HD, which emphasizes continuous net fluid removal, I-HDF provides a cyclical pattern of volume restoration followed by gentle removal. This dynamic helps stabilize intravascular volume and may reduce hemodynamic stress. Although both I-HDF and online HDF utilize convective transport to enhance solute clearance, their implementation differs markedly. A side-by-side comparison of these two modalities is provided in Table 1, highlighting the differences in fluid volume, source, and machine requirements. Figure 1 provides a schematic comparison of these modalities, highlighting the fundamental differences in fluid movement and circuit configuration between I-HDF (intermittent infusion and ultrafiltration phases) and pre/post-dilution online HDF.

## 3. Core Clinical Benefits of I-HDF: Hemodynamic Stability, Organ Protection, and Toxin Clearance

### 3.1. Significant Reduction in IDH and Hemodynamic Stabilization

#### 3.1.1. Reducing IDH Incidence and Severity

IDH is one of the most common and clinically significant complications during conventional HD, with an estimated incidence of around 11% per treatment session [4]. It often results from an imbalance between the rate of plasma volume loss and the body’s ability to compensate. Conventional HD with continuous ultrafiltration can lead to a rapid decline in circulating blood volume (BV) and reflex vasodilation [18]. When the plasma volume reduction exceeds the plasma refilling capacity, IDH is likely to occur. This leads to several negative consequences, including inadequate dialysis, vascular access thrombosis, cardiovascular events, and cumulative end-organ ischemia due to repeated hypoperfusion [19,20,21,22].

I-HDF is designed to stabilize circulation by applying a volume-centered treatment model. During treatment, the rapid dialysate infusion increases preload and cardiac output, helping prevent a sudden drop in blood pressure [23].Clinical trials have consistently shown that I-HDF reduces IDH incidence and the need for intervention. A multicenter trial by Koda et al. [13] involving 77 patients prone to IDH showed that switching from conventional HD to I-HDF significantly reduced the need for IDH-related interventions, such as saline boluses or leg elevation. The median number of interventions per patient per month dropped from 4.5 to 3.0 (*p* < 0.01). A single-center study by Fan et al. [24], involving 30 patients with frequent IDH, further confirmed that I-HDF significantly reduced the magnitude of blood pressure decline, particularly in the early phase of treatment. Moreover, I-HDF enabled better achievement of the target ultrafiltration volume and reduced premature termination due to hypotension.

Longer-term effects were evaluated in a multicenter, parallel-group study by Mineshima et al. [14], comparing I-HDF with pre-dilution online HDF, a modality commonly used in parts of Asia due to lower blood flow rates [25]. While short-term blood pressure changes did not differ significantly between groups during the first week, both I-HDF and HDF showed improved blood pressure stability over time compared to HD. From the 13th week onward, the I-HDF group demonstrated a significantly lower rate of IDH events requiring clinical intervention, suggesting that the hemodynamic benefits of I-HDF may accumulate with sustained use over a six-month period.

#### 3.1.2. Enhancing Volume Homeostasis and Autonomic Balance

From a physiological perspective, IDH results from an imbalance between fluid removal and compensatory mechanisms involving vascular tone, heart rate, cardiac contractility, and splanchnic blood flow redistribution. These are regulated primarily by the sympathetic nervous system and the renin–angiotensin–aldosterone system [21,26,27]. Hemodynamic monitoring data consistently show that I-HDF offers advantages over conventional HD in this regard. Continuous BV monitoring further confirms that patients receiving I-HDF experience a slower decrease in circulating BV. A comparative study [10] showed that the rate of time-averaged BV decrease during I-HDF is significantly lower than that during conventional HD, despite comparable total fluid removal. This indicates that, compared to conventional HD, I-HDF facilitates more effective transcapillary fluid and solute mobilization from the extravascular to intravascular space, resulting in a higher plasma refilling rate (PRR), which is associated with reduced IDH [28]. These findings were supported by a prospective observational study from Ookawara et al. [29], which found similar trends in patients at a high risk of IDH. Heart rate measurements indicate that as intravascular volume decreases, HD patients often exhibit compensatory tachycardia, i.e., a marker of heightened sympathetic activation. In contrast, patients undergoing I-HDF are able to maintain a more stable and lower heart rate [13]. This suggests that I-HDF reduces the burden on compensatory systems and blunts sympathetic overactivity. Excessive sympathetic activation has been proposed as a potential upstream trigger for the afferent vagal stimulation that initiates the Bezold–Jarisch reflex, a paradoxical cardiovascular response associated with sudden intradialytic hypotension [30,31]. By attenuating sympathetic overactivity, I-HDF may prevent the initiation of BJR and thereby reduce the risk of acute circulatory collapse during dialysis.

#### 3.1.3. Pausing Ultrafiltration as a Co-Contributor

While the role of dialysate infusion in promoting hemodynamic stability is well documented, some researchers have proposed that the benefits may also stem from the brief interruption of fluid removal. In a crossover study [32], Igarashi et al. compared standard I-HDF (200 mL infusion every 30 min) with a modified HD–rest pause (HD-RP) protocol, where ultrafiltration was paused for 80 s every 30 min without any infusion. Surprisingly, the HD-RP protocol showed even smaller blood pressure and volume reductions than I-HDF. This suggests that part of I-HDF’s benefit may be attributed to the temporary suspension of ultrafiltration itself, in addition to the effects of volume infusion.

**Figure 2:**
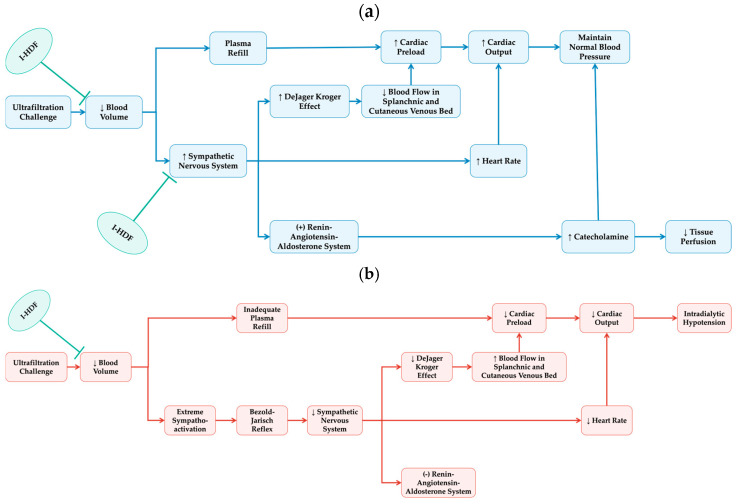
Mechanistic role of I-HDF in hemodynamic regulation during hemodialysis. (**a**) Compensated response (blue): During conventional HD, ultrafiltration reduces blood volume, triggering compensatory mechanisms such as plasma refilling and sympathetic activation to preserve blood pressure. I-HDF supports this phase by intermittently infusing dialysate to increase intravascular volume and enhance plasma refill. This approach reduces the need for excessive sympathetic drive, helping maintain hemodynamic stability and prevent progression to decompensation. (**b**) Decompensated response leading to intradialytic hypotension (red): When compensatory mechanisms fail, excessive sympathetic activation may paradoxically trigger the Bezold–Jarisch reflex, leading to bradycardia and hypotension. I-HDF helps mitigate intradialytic hypotension by restoring circulating volume and supporting systemic perfusion.

Overall, the evidence supports that I-HDF provides more stable fluid removal and better hemodynamic control compared to conventional HD. As illustrated in Figure 2, I-HDF achieves this by intermittently infusing ultrapure dialysate, which cyclically expands intravascular volume and enhances plasma refilling. During the compensated phase of hemodynamic response, this mechanism reduces reliance on excessive sympathetic activation, thereby helping preserve cardiovascular stability. The cyclic volume expansion of I-HDF can mitigate the hemodynamic stress imposed by ultrafiltration, thus offering a viable strategy to reduce IDH frequency and severity. For patients prone to IDH, I-HDF represents a promising therapeutic approach that not only reduces the need for intervention, but also improves treatment continuity and fluid management.

### 3.2. Improvement of Organ and Microcirculatory Perfusion

IDH can result in serious ischemic consequences across multiple organs. It has been associated with myocardial stunning and impaired cardiac contractility, cerebral hypoperfusion and ischemic brain injury, the loss of residual kidney function, intestinal ischemia, and critical limb ischemia. In severe cases, these complications may lead to patient mortality [33,34,35,36]. By improving systemic perfusion, I-HDF may reduce the frequency and severity of these ischemic events that are commonly observed during conventional HD.

#### 3.2.1. Preserving Cardiac Function

Patients with chronic heart failure or left ventricular hypertrophy are especially sensitive to volume shifts during dialysis. Rapid fluid removal may exceed cardiac tolerance, triggering hypotensive episodes and further burdening cardiac function. In addition to volume changes, neurohormonal mechanisms—particularly activation of the sympathetic nervous system —also play a central role in the progression of heart failure [37]. Therefore, stabilizing both fluid balance and sympathetic activity during dialysis may confer cardioprotective benefits. I-HDF offers a therapeutic alternative for this population. In a study by Saito et al. [38], five MHD patients with reduced cardiac function were switched from conventional HD to I-HDF after six months. Their cardiothoracic ratio (CTR) improved, suggesting a reduction in volume overload. Echocardiographic monitoring showed increases in left ventricular ejection fraction, reductions in left ventricular end-diastolic diameter, and significant decreases in end-systolic diameter (*p* < 0.05), indicating better myocardial contractility. These findings counter early concerns that dialysate infusion may overload the heart [39]. Instead, because I-HDF maintains a stable net ultrafiltration rate and avoids abrupt drops in preload, it supports cardiac function, reduces myocardial ischemia, and enhances hemodynamic stability in patients with cardiac dysfunction [40,41].

#### 3.2.2. Improving Hepatic Oxygenation

Beyond the heart, I-HDF may also enhance oxygen delivery to other organs. A study comparing symptomatic and asymptomatic IDH patients found that hepatic regional oxygen saturation (rSO_2_) declined more sharply before IDH onset in symptomatic individuals [42]. This implies that hepatic rSO_2_ may serve as a sensitive indicator of perfusion-related stress. Ookawara et al. [29] monitored both cerebral and hepatic rSO_2_ during HD and I-HDF. Cerebral rSO_2_ stayed mostly stable during both treatments, though slight increases occurred after infusions in the second half of I-HDF sessions. Hepatic rSO_2_, however, showed different trends: in HD, it dropped early, partially recovered mid-treatment, then declined again. In contrast, during I-HDF, hepatic rSO_2_ rose in a wave-like pattern with each infusion, peaking after the final infusion. The degree of hepatic rSO_2_ improvement was negatively correlated with the relative BV (ρ = −0.394, *p* < 0.001) and positively correlated with the infusion volume-to-BV ratio before each bolus (ρ = 0.387, *p* < 0.001). This suggests that patients with more favorable volume status, or those receiving proportionally larger infusions, experience greater hepatic oxygenation. Although long-term outcomes such as cognitive function or myocardial stunning were not evaluated, better tissue oxygenation may contribute to prevent repeated ischemia–reperfusion injury.

#### 3.2.3. Enhancing Peripheral Circulation

I-HDF may also enhance peripheral tissue perfusion. In a study by Nagao et al. [32], patients were grouped by the presence of arteriosclerosis obliterans (ASO). Blood flow in the dorsal foot, measured by laser Doppler flowmetry, did not improve significantly during HD. In contrast, I-HDF increased lower limb perfusion by 29.9% in non-ASO patients and 10.1% in the ASO group. Interestingly, when I-HDF was combined with CO_2_ mist therapy, blood flow improved further in the ASO group. Conventional HD has been shown to impair peripheral microcirculatory blood flow [43]. Saito et al. [38] observed that PRR was higher during I-HDF and that peripheral perfusion, measured at the earlobe, improved alongside better hemodynamic profiles and lower sympathetic activation. These results support the view that intermittent infusions in I-HDF not only protect vital organs from ischemia but also improve microvascular flow and peripheral tissue oxygenation. This may help prevent phenomena such as the De Jager–Krogh effect, in which venous pooling during ultrafiltration impairs central circulation [44].

In summary, I-HDF has been shown to confer measurable benefits in preserving organ and microcirculatory perfusion during dialysis. These improvements likely result from more stable hemodynamics and a fluid trajectory that better mimics physiological patterns. Current research has focused mainly on several single-organ observations but still lacks exploration. For example, while conventional HD has been associated with ischemic injury to the spleen [45] and intestines [46,47], the impact of I-HDF on these organs remains unexplored. Nonetheless, the cross-organ responses raise the possibility that I-HDF may confer broader systemic benefits, such as improved endotoxin clearance and better maintenance of immune homeostasis, while this hypothesis remains speculative and warrants further investigation.

### 3.3. Enhancement of Toxin Removal and Membrane Performance

#### 3.3.1. Promoting Middle Molecule Removal

I-HDF may improve solute clearance efficiency by enhancing peripheral perfusion through cyclic fluid infusions. This enhancement promotes the transcapillary transport of water and solutes from the extravascular space into the circulation, thereby supporting more effective toxin removal. Essentially, I-HDF is a conventional HD technique enhanced with intermittent infusions, combining both diffusive and convective transport mechanisms. Convective therapies are particularly effective in removing middle-molecular-weight uremic toxins [48,49]. Although the total replacement volume is modest (approximately 1.4 L), I-HDF has demonstrated selective clearance advantages for specific solutes, which may translate into meaningful clinical benefits.

For instance, in a retrospective study, Takahashi et al. [32] observed the significant relief of pruritus symptoms in 15 patients who switched from conventional HD to I-HDF. The authors hypothesized that this improvement was associated with better clearance of pruritogenic toxins such as β_2_-microglobulin (β_2_-MG) [50,51]. Although high-volume online HDF typically provides superior clearance of middle molecules, I-HDF has demonstrated meaningful improvements compared to conventional HD in this area.

Several studies have directly compared the solute clearance profiles of I-HDF and conventional HD. In a trial by Mineshima et al. [52], I-HDF achieved significantly higher clearance rates of β_2_-MG and α_1_-microglobulin (α_1_-MG), both of which are established middle-molecular-weight toxins. Interestingly, the clearance of small solutes like urea and creatinine was slightly lower in I-HDF with no significant differences. An in vitro study [53] investigating the impact of infusion parameters on solute removal helps explain this finding. It showed that increasing the infusion rate, volume, or frequency improved β_2_-MG clearance, but slightly reduced urea clearance. This may be due to transient blood dilution, which weakens the concentration gradient necessary for diffusion. In contrast, middle-molecular-weight toxins benefit from stronger convective transport. Differences in membrane properties also play a role. In regions where relatively larger-pore-membrane hemodialyzers are not covered by insurance, I-HDF is often performed with smaller-pore-membrane hemodiafiltraters, which could slightly limit the diffusion-based clearance of small molecules.

#### 3.3.2. Preserving Dialyzer Performance

Another important feature of I-HDF is its ability to maintain membrane performance throughout treatment. An intermittent high-flushing effect was observed in in vitro experiments. Compared with the other two dialysis methods, the degree of membrane fouling decreased with the cyclic backflushing [54]. In conventional HD, middle-molecular-weight solutes such as α_1_-MG tend to accumulate on the membrane surface during ultrafiltration, leading to fouling and reduced permeability over time. This decreases solute clearance during long sessions [55]. I-HDF addresses this issue through periodic backflushing with ultrapure dialysate, which helps remove protein deposits. For example, one analysis reported that α_1_-MG clearance dropped to around 17% in conventional HD but remained at approximately 21% throughout I-HDF treatment [52]. This backflush effect allows for I-HDF to maintain more consistent clearance of protein-bound and mid-sized toxins across the session.

#### 3.3.3. Minimizing Albumin Loss

Hypoalbuminemia is a strong and independent predictor of cardiovascular events and all-cause mortality in dialysis patients [56,57]. While improving the clearance of middle- and large-molecular-weight uremic toxins has a greater overall impact on survival than minimizing protein loss alone [58], reducing unnecessary albumin leakage remains a clinically relevant goal, particularly when clearance efficiency is comparable between modalities.

I-HDF appears to offer a favorable balance between convective clearance and protein preservation. In a multicenter trial, Mineshima et al. [14] evaluated I-HDF (~1.4 L infusion) against pre-dilution HDF (~44.9 L replacement volume). I-HDF showed comparable removal efficiency on medium-sized toxins, with significantly reduced albumin leakage. Some retrospective data [32] have reported increased serum albumin levels following transition from conventional HD to I-HDF for three months. Additionally, bioimpedance data showed a rise in body fat weight, suggesting improved nutritional status [59]. While these observations lack controlled design and should be interpreted with caution, whether I-HDF influences albumin synthesis or broader nutritional markers remains to be investigated.

Overall, I-HDF offers a unique toxin clearance profile that differs from both conventional HD and pre-dilution HDF. Compared to HD, the added convective component of I-HDF improves the removal of middle-molecular-weight toxins such as β_2_-MG and α_1_-MG. Compared to pre-dilution HDF, I-HDF achieves similar solute removal with markedly lower albumin loss. For the management of uremic toxicity—particularly in vulnerable patient populations—I-HDF represents a balanced, physiologically informed approach that may optimize both toxin removal and clinical safety.

## 4. Survival Advantage of I-HDF

Whether I-HDF can improve patient survival compared to conventional HD remained an open question until recent years. To investigate this, Abe et al. [60] conducted a large retrospective cohort study using data from the national dialysis registry in Japan. This study included 210,574 maintenance HD patients over a two-year period, of whom 15,551 were receiving I-HDF. After adjusting for relevant clinical factors, the researchers found that patients treated with I-HDF had a significantly lower risk of all-cause mortality compared to those on standard HD.

To further understand the survival impact of infusion volume, the I-HDF group was divided into two subgroups: low-volume (<1.2 L per session) and high-volume (≥1.2 L per session) treatment. The results showed that patients in the high-volume group had better survival outcomes than those receiving low-volume I-HDF or conventional HD. More importantly, cardiovascular mortality was lower in the high-volume I-HDF subgroup. These findings suggest a dose–response relationship, indicating that achieving sufficient infusion volume is likely important for long-term survival benefits. Subgroup analysis showed that certain patient populations—such as women, elderly individuals (≥70 years), those with a longer dialysis vintage (≥72 months), lower BMI (<22), lower systolic blood pressure (<150 mmHg), lower serum albumin (<3.6 g/dL), and no history of diabetes or cardiovascular disease—may benefit most from I-HDF treatment.

**Figure 3:**
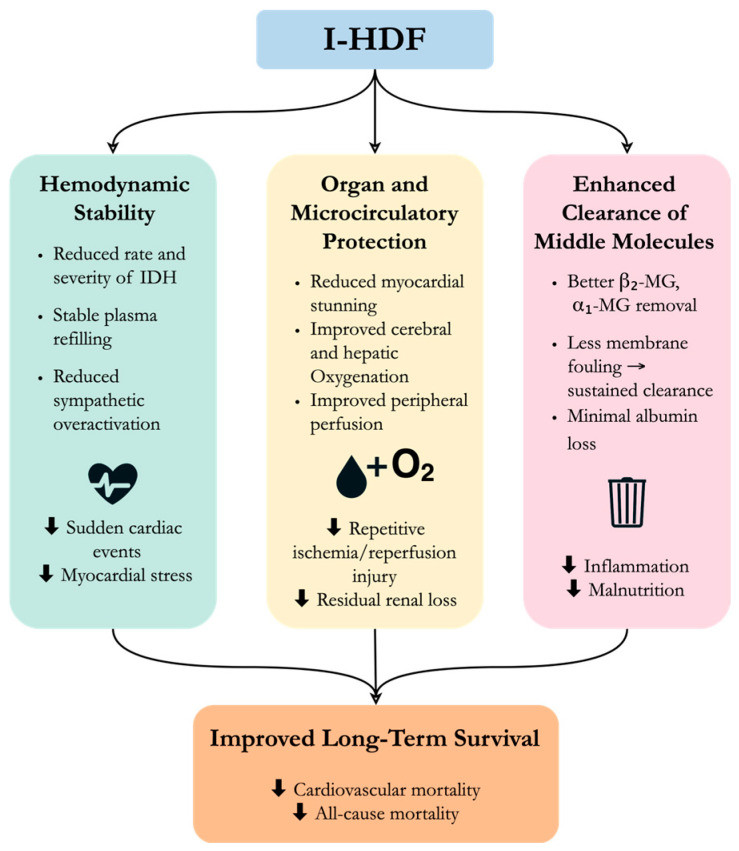
Proposed mechanisms by which intermittent infusion hemodiafiltration (I-HDF) may improve survival. By stabilizing hemodynamics, protecting organ perfusion, and enhancing middle-molecule clearance, I-HDF may reduce cardiovascular stress, ischemic injury, inflammation, etc., contributing to better long-term outcomes.

What might explain the observed survival benefit of I-HDF? Although these findings are based on observational data and cannot confirm causality, there are several possible mechanisms (Figure 3). First, I-HDF stabilizes hemodynamics and reducing the incidence of IDH, a well-known predictor of mortality in dialysis patients [61]. Second, more stable blood pressure during dialysis helps protect vital organs from repeated ischemia and support better organ perfusion. Better hemodynamic control also supports achievement of the target dry weight, which could help reduce chronic volume overload, thereby lowering cardiac strain [62,63]. Third, I-HDF is effective at removing β_2_MG and other middle-molecular-weight toxins. Since elevated β_2_MG levels are linked to chronic inflammation and poor outcomes [64,65], better clearance may contribute to improved survival.

It is worth noting that when analyzing the entire I-HDF group as a whole, no significant difference in cardiovascular mortality was observed compared to HD. This may be due to variability in infusion protocols and total substitution volume among different patients. These findings highlight the importance of personalized treatment strategies in I-HDF, which will be discussed in the next section.

## 5. Individualized Treatment in I-HDF

Although current evidence supports the clinical efficacy of I-HDF, variations in study results suggest that treatment response may differ between patients. For example, in a crossover randomized controlled trial by Mineshima et al. [52], no significant differences were observed in blood pressure control or the frequency of clinical interventions between I-HDF and conventional HD. Similarly, Ookawara et al. [29] reported no significant changes in average blood pressure before, during, or after dialysis between the two modalities. In contrast, Koda et al. [13] found that I-HDF stabilized the progressive decline in systolic blood pressure during dialysis. This discrepancy may be explained by differences in patient selection. In Koda’s study, all participants were prone to IDH, and thus blood pressure indicators were more likely to benefit from the circulatory stabilization offered by I-HDF. These findings highlight that patient-specific characteristics significantly influence treatment response. As such, tailoring I-HDF parameters to individual conditions may enhance both safety and effectiveness. This personalized approach is an important step toward realizing the goal of precision dialysis.

### 5.1. Initiation Criteria

A study by Eiki and Otake [39] examined why some patients do not respond well to I-HDF and provided suggestions for clinical decision-making. They proposed that I-HDF can be considered when only one of the following conditions is present: (1) severe arteriosclerosis obliterans (ASO), (2) more than mild valvular heart disease, or (3) cardiothoracic ratio (CTR) ≥ 55% in men or ≥60% in women, a rapid increase in CTR, or persistent systolic blood pressure > 180 mmHg during dialysis. However, if two or more of these factors are present simultaneously, I-HDF initiation may not be advisable.

### 5.2. Infusion Volume

Fan et al. [24] reported that one of thirty patients developed chest discomfort during a 250 mL infusion, suggesting that excessive infusion volumes can cause transient symptoms. Therefore, individualized adjustment of infusion volume is necessary. Dry weight is a practical estimate of circulating blood volume and can guide volume prescriptions. In a study by Otsubo et al. [66], patients with a dry weight over 52 kg experienced more stable post-dialysis blood pressure—on average 3 mmHg higher—when treated with 200 mL infusions compared to 100 mL. In contrast, patients under 52 kg did not benefit more from the larger volume, indicating that smaller patients may tolerate lower doses better. This weight cutoff is based on the estimation that 200 mL equals approximately 5% of circulating blood volume in a 52 kg individual. Moreover, higher infusion volumes in larger patients were associated with reduced heart rate elevation, indicating lower sympathetic activation. Based on these findings, an infusion volume corresponding to 2.5–5% of the estimated blood volume is considered appropriate for safe and effective treatment.

### 5.3. Infusion Interval

While the standard I-HDF protocol delivers infusions every 30 min [13], this interval may need adjustment based on clinical context. Ideally, the infusion interval should be tailored to the patient’s actual hemodynamic status rather than applied uniformly. For instance, in patients at a high risk of early-session IDH or rapid blood volume loss, more frequent and smaller infusions may offer better hemodynamic stability. Conversely, patients who tolerate the early phase but develop hypotension later in the session may benefit from increasing infusion frequency during the second half. Instead of fixed intervals, the real-time monitoring of blood volume may allow for infusions to be triggered when volume falls below a predefined threshold. This approach ensures that fluid is delivered precisely when needed. Additionally, administering infusions during periods of low blood volume and high plasma solute concentration may improve solute clearance by enhancing convective transport efficiency [53,67]. Therefore, optimizing infusion timing can contribute to both better hemodynamic control and more effective toxin removal.

Despite promising findings from observational studies and small clinical trials, further large-scale investigations are needed to define standardized protocols for individualized I-HDF treatment. These future studies should clarify optimal patient selection, volume dosing, and infusion timing strategies to support the evidence-based personalization of therapy.

## 6. Future Directions

### 6.1. Toward Precision and Accessibility in I-HDF

Integrating smart technologies into dialysis systems is poised to advance the precision of individualized I-HDF therapy significantly. Current dialysis machines are already capable of continuously monitoring dynamic changes in BV, and some can estimate surrogate parameters such as relative blood pressure or cardiac index. Building on these capabilities, closed-loop feedback systems have been developed: Hamada et al. [68] have used a blood volume change-guided ultrafiltration control (BV-UFC) system to dynamically control the ultrafiltration rate during dialysis in HD patients. Compared with the control phase without this system, BV-UFC significantly reduced the number of IDH episodes per dialysis session. However, the proportion of dialysis sessions with at least one IDH event did not differ significantly between the two groups. This suggests that using a blood volume change-guided ultrafiltration control system may help alleviate recurrent hypotensive episodes rather than completely prevent IDH. If such feedback systems were applied to guide real-time parameter adjustment in I-HDF, they could potentially regulate not only ultrafiltration rates, but also optimize infusion patterns dynamically according to the patient’s evolving physiological status. This real-time adaptability may offer better protection against IDH than when such systems are applied to HD alone.

In addition, novel monitoring technologies are being developed to provide multidimensional physiological feedback, e.g., tools such as near-infrared spectroscopy for tissue oxygenation and laser Doppler flowmeters for peripheral blood flow. For example, a decline in hepatic rSO_2_ or peripheral perfusion could be seen as early warning signs of impending IDH and trigger infusion interventions. These data streams can be incorporated into algorithm-driven systems that move beyond fixed infusion schedules and deliver dynamically optimized I-HDF therapy tailored to each patient’s needs.

In short, personalized treatment is key to unlocking the full potential of I-HDF therapy. Evidence-based strategies—such as adjusting infusion volume based on dry weight or tailoring infusion frequency based on blood pressure—are both practical and clinically feasible. At the same time, advanced personalized models integrating real-time monitoring with predictive algorithms are emerging as a cutting-edge direction in translational medicine innovation [69,70,71]. A stepwise implementation of individualized approaches can enhance the safety of I-HDF, improve efficacy, and ultimately elevate treatment tolerability and long-term patient outcomes.

### 6.2. Potential Role of I-HDF Under Policy and Resource Constraints

The selection of dialysis modality is often influenced by local healthcare policies and insurance reimbursement systems [72,73]. In certain regions, policy limitations may prevent patients from accessing the most clinically appropriate treatment. For instance, in some healthcare systems, hypotension is not an indication for online HDF treatment and cannot be reimbursed by medical insurance. Patients with intradialytic hypotension are more likely not to choose HDF for dialysis due to financial reasons, which limits patients’ opportunities to achieve optimal treatment outcomes. Although I-HDF generates convection during backfiltration, it does not qualify as HDF based on the definition of convective volume. Consequently, I-HDF is not categorized or billed as HDF in these regions and is not subject to the same reimbursement restrictions. In this context, I-HDF provides a practical and accessible alternative for managing IDH in patients who are otherwise excluded from receiving HDF due to current insurance frameworks and broader healthcare policy limitations.

I-HDF can also be implemented in resource-limited environments through innovative manual infusion techniques. For example, intermittent manual saline infusions may be administered, with the total ultrafiltration volume set as “the patient’s actual fluid removal requirement + total infused volume” [15]. This method has been shown to be safe and effective in preventing IDH as machine-based I-HDF. In regions where transplantation opportunities and healthcare infrastructure remain limited, advancing dialysis strategies plays a pivotal role in improving patient outcomes [74,75]. I-HDF has the potential to enhance clinical outcomes through its technical advancements while also overcoming resource constraints through adaptable implementation strategies, making it possible to achieve I-HDF’s advantages for dialysis patients in a wide range of healthcare environments.

## 7. Discussion

This review outlines the physiological rationale, technical considerations, and clinical applications of I-HDF. To contextualize the current body of evidence, we compiled a structured overview of key clinical studies evaluating I-HDF, presented in Table 2. This summary highlights the study designs, patient populations, and main findings across a range of clinical endpoints, including hemodynamic stabilization, toxin clearance, and survival.

It is important to note that most of the available clinical data on I-HDF originate from Japan, where this modality is most widely implemented within a highly standardized dialysis framework. While the Japanese experience offers valuable insights—particularly in light of the country’s globally recognized end-stage renal disease outcomes [76]—the geographic concentration of evidence inevitably limits its generalizability. Variations in dialysis protocols, patient characteristics, and reimbursement structures across regions may affect the feasibility and effectiveness of I-HDF elsewhere. We have explicitly acknowledged this limitation and emphasized the need for future multinational and multicenter studies to validate the broader applicability of I-HDF in diverse healthcare settings.

Furthermore, although existing studies consistently report favorable outcomes with I-HDF—including improvements in blood pressure regulation, plasma refilling, and middle-molecule clearance—the strength of the evidence must be interpreted with caution. Many studies involve small sample sizes, short follow-up periods, and non-randomized designs. The largest survival analysis [60], to date, while compelling due to its scale, is retrospective and thus subject to confounding and selection bias. Only a limited number of trials directly compare I-HDF to other high-efficiency modalities, further limiting conclusions about its relative superiority. We, therefore, refrained from overstating mechanistic claims and have clearly acknowledged the narrative nature and limitations of the current evidence base throughout the manuscript.

In summary, while the physiological rationale and emerging clinical data for I-HDF are promising, additional high-quality research is necessary to establish its comparative effectiveness, optimal implementation strategies, and long-term benefits across heterogeneous patient populations and dialysis environments.

## 8. Conclusions

I-HDF is an original and practical advancement in the field of blood purification. By integrating cyclic dialysate backfiltration into standard HD, I-HDF offers a triple benefit: improving hemodynamic stability, enhancing organ perfusion, and increasing the clearance of middle-molecular-weight toxins without excessive albumin loss. These features directly address two major limitations of conventional HD–IDH and suboptimal toxin removal. Large-scale observational data indicate that I-HDF may contribute to improved long-term survival, especially in patients receiving higher infusion volumes. Importantly, these clinical benefits may be achieved without the infrastructure demands or policy restrictions, making I-HDF especially valuable in health systems with constrained resources or limited transplantation access. The development of individualized infusion strategies, guided by parameters such as dry weight and real-time blood volume monitoring, is crucial for maximizing the efficacy and safety of I-HDF. Moreover, integrating intelligent feedback systems and emerging biosignal monitoring tools may further advance precision dialysis. Despite its promising potential, more validation through large-scale, multicenter studies is required. As evidence continues to accumulate, I-HDF is likely to play an increasingly important role in enhancing the quality of life and long-term outcomes of patients undergoing dialysis.

## Figures and Tables

**Table 1:** Key technical differences between intermittent infusion HDF (I-HDF) and online HDF.

Aspect	I-HDF	Online HDF
Infusion Timing	Intermittent (e.g., every 30 min)	Continuous throughout the dialysis session
Infusion Volume	Typically 200 mL per infusion; total ~1.4 L per session (default mode)	Typically ≥20% of total processed blood volume [15]
Infusate Source	Ultrapure dialysate (also used as the standard dialysis fluid in conventional HD) serves as the primary reinfusion fluid. Saline may be used for manual infusion. Microbiological standards [16]: endotoxin concentration < 0.001 EU/mL; total viable microbial count < 0.1 CFU/mL.	Online-produced sterile substitution fluid Microbiological standards [17]: endotoxin concentration < 0.03 EU/mL; total viable microbial count < 0.1 CFU/mL.
Machine Requirements	Standard HD machine with rapid infusion mode; specialized HD machine with backfiltration capability; manual infusion (rarely used in clinical setting)	Specialized HDF machine with online substitution module

**Table 2:** Representative clinical studies on intermittent infusion hemodiafiltration (I-HDF).

Study (Author, Year)	Design	Sample Size/Comparison	Key Findings
Mineshima and Eguchi, 2013 [10]	Multicenter, crossover trial	*n* = 20 I-HDF versus HD	I-HDF improved peripheral circulation and plasma refilling.
Koda et al., 2017 [13]	Multicenter, crossover trial (4 + 4 weeks)	*n* = 77 I-HDF versus HD Patients prone to hypotension	I-HDF reduced IDH events, maintained higher systolic BP, and blunted heart rate elevation. Most beneficial in older patients and those with higher interdialytic weight gain.
Mineshima et al., 2017 [14]	Multicenter, parallel-group comparative trial (26 weeks)	*n* = 36 I-HDF versus predilution online HDF	No difference in clinical symptoms or quality of life between groups. I-HDF achieved significantly less albumin loss.
Mineshima et al., 2019 [52]	Multicenter, randomized controlled crossover trial (14 + 14 + 14 weeks)	*n* = 64 I-HDF versus HD	I-HDF improves removal of medium-size uremic toxins. Albumin leak was less with I-HDF.
Saito et al., 2019 [38]	Prospective observational study (6 + 6 months)	*n* = 5 I-HDF versus HD Patients with cardiac hypofunction	I-HDF facilitates safe fluid removal in cardiac dysfunction patients. I-HDF may aid heart failure management by enhancing plasma refilling and avoiding hypoperfusion.
Eiki and Otake, 2019 [39]	Retrospective comparative study	*n* = 36 I-HDF: effective group versus ineffective group	Not all patients respond equally to I-HDF. I-HDF was most effective in patients with frequent IDH and manageable volume status.
Otsubo et al., 2019 [66]	Prospective observational study (2 + 2 weeks)	*n* = 77 Infusion volume: 100 mL versus 200 mL	Better to determine the amount of infusion based on patient’s dry weight
Abe et al., 2024 [60]	Nationwide cohort study (2 years)	*n* = 210,574 I-HDF versus HD	I-HDF is linked to modest survival gains. I-HDF has a dose-dependent benefit, though causality needs confirmation.
Ookawara et al., 2025 [29]	Prospective observational study	*n* = 25 I-HDF versus HD	I-HDF improves hepatic oxygenation and stabilizes blood volume during infusion phase.

## Data Availability

Not applicable.

## Abbreviations

The following abbreviations are used in this manuscript:

I-HDF	Intermittent infusion hemodiafiltration
HD	Hemodialysis
IDH	Intradialytic hypotension
BV	Blood volume
MHD	Maintenance hemodialysis
HDF	Hemodiafiltration
SBP	Systolic blood pressure
PRR	Plasma refilling rate
CTR	Cardiothoracic ratio
rSO_2_	Regional oxygen saturation
ASO	Arteriosclerosis obliterans
β_2_-MG	β_2_-microglobulin
α_1_-MG	α_1_-microglobulin
